# Molecular identification of zoonotic and livestock-specific *Giardia*-species in faecal samples of calves in Southern Germany

**DOI:** 10.1186/1756-3305-6-346

**Published:** 2013-12-10

**Authors:** Julia Gillhuber, Louise Pallant, Amanda Ash, RC Andrew Thompson, Kurt Pfister, Miriam C Scheuerle

**Affiliations:** 1 Comparative Tropical Medicine and Parasitology, Faculty of Veterinary Medicine, Ludwig-Maximilians-Universität München, Leopoldstr. 5, Munich 80802, Germany; 2 School of Veterinary and Biomedical Sciences, Murdoch University, Perth, Western Australia, Australia

**Keywords:** PCR, Diarrhoea, Protozoan, *Giardia* assemblages, Cattle, *Giardia duodenalis* morphological group

## Abstract

**Background:**

*Giardia*-infection in cattle is often subclinical or asymptomatic, but it can also cause diarrhoea. The livestock-specific species *Giardia bovis* is the most frequently observed in cattle, however, the two zoonotic species *Giardia duodenalis* and *Giardia enterica* have also been found. Therefore calves are thought to be of public health significance. The aim of this study was to obtain current data about the frequency of the different *Giardia*-species in calves in Southern Germany.

**Findings:**

Faecal samples of calves (diarrhoeic and healthy) in Southern Germany, diagnosed *Giardia*-positive by microscopy, were characterised by multi-locus PCR and sequencing.

Of 152 microscopically *Giardia*-positive samples 110 (72.4%) were positive by PCR and successfully sequenced. *G. bovis* (Assemblage E) was detected in 101/110 (91.8%) PCR-positive samples, whilst *G. duodenalis* (Assemblage A) was detected in 8/110 (7.3%) samples and a mixed infection with *G. duodenalis* and *G. bovis* (Assemblage A+E) was identified in 1/110 (0.9%) samples. The sub-genotypes A1, E2 and E3 were identified with the β-giardin and the glutamate dehydrogenase genes. In the majority of diarrhoeic faecal samples a co-infection with *Cryptosporidium* spp. or *Eimeria* spp. was present, however, there were some in which *G. bovis* was the only protozoan pathogen found.

**Conclusions:**

The results suggest that there is potentially a risk for animal handlers as calves in Southern Germany are, at a low percentage, infected with the zoonotic species *G. duodenalis.* In addition, it was found that *G. bovis* was the only pathogen identified in some samples of diarrhoeic calves, indicating that this parasite may be a contributing factor to diarrhoea in calves.

## Findings

### Background

Worldwide the protozoan *Giardia* spp. is one of the most common intestinal parasites in humans (reviewed in [1,2]) and also a frequent enteric parasite in animals including companion animals, livestock and wildlife [2]. According to Monis *et al*. [3] there are eleven species within the genus *Giardia*. Six of them, formally known as Assemblages A-G of the *Giardia duodenalis* morphological group, are genetically but not morphologically distinguishable. They can infect humans and mammals, with some being host specific and others having low host specificity.

*Giardia-*infection in cattle is often subclinical or asymptomatic, but this infection can also cause symptoms including acute or chronic diarrhoea, reduced weight gain and ill thrift in young calves [4,5]. Although the prevalence of *Giardia* in cattle around the world varies considerably (reviewed in [5,6]), longitudinal studies have shown cumulative infection rates in calves of 100% [7,8]. The two zoonotic species *G. duodenalis* (Assemblage A) and *G. enterica* (Assemblage B) and the livestock-specific species *G. bovis* (Assemblage E) are able to infect cattle with *G. bovis* being found most frequently followed by *G. duodenalis*[9-13]. Therefore, calves are thought to be of public health significance both as a source of waterborne outbreaks of giardiasis in humans and as a risk to in-contact animal handlers [2,14].

Current data on the occurrence of the different *Giardia* species in German calves is only available for 2–16 week-old calves from farms around Berlin. In that study (15) a commercially available monoclonal antibody-based ELISA was used and *Giardia* was detected in 100% of the farms and 51.2% of the animals sampled. Subsequent molecular characterisation ascertained *G. bovis* (Assemblage E) was the most common species present, but infections with *G. duodenalis* (Assemblage A) and mixed infections of *G. duodenalis* and *G. bovis* (Assemblage A+E) were also found [15].

Thus, the aim of this study was to obtain current data about the frequency of the different *Giardia* species in calves of a wider range of age in Southern Germany.

### Methods

#### Samples

Faecal samples of calves from the southern federal states of Germany, Bavaria and Baden-Württemberg, were sent to the Diagnostic Laboratory of Comparative Tropical Medicine and Parasitology, LMU Munich, Germany for microscopy analysis. *Giardia* spp., *Cryptosporidium* spp. and *Eimeria* spp. were detected using the carbolfuchsin-stained direct faecal smear [16] and the merthiolate iodine formaldehyde concentration (MIFC) with the addition of Lugol’s solution [17]. Samples from 152 calves between 3 and 130 days of age (mean age: 50.7 days, n = 138) were diagnosed *Giardia*-positive by the MIFC-method between June 2011 and January 2013 and stored at −20°C. In February 2013 these samples were preserved in 70% ethanol and sent to the School of Veterinary and Life Sciences, Murdoch University, Australia, for molecular characterisation.

#### DNA extraction

DNA was extracted from faecal samples using the Maxwell® 16 Tissue DNA Purification Kit (Promega, Madison, USA) with the Maxwell® 16 Instrument (Promega). In addition to the recommended protocol, 1 μl of the final elution was further diluted by adding 4 μl of Water-ultra pure grade (Fisher Biotech Perth, Australia). Both neat and dilute templates were used in PCRs.

#### PCR amplification

For the amplification of the 18S rRNA gene and the β-giardin gene a nested PCR was carried out and for the amplification of the glutamate dehydrogenase (GDH) gene a semi-nested PCR was performed. Details of primers and cycling conditions are listed in Table 1.

**Table 1:** PCR conditions and primers

**Target gene**	**Number of reaction**	**Length of amplification (bp)**	**Primer**	**Cycle condition**	**Reaction volume**	**Reference**
18S rRNA	Primary reaction	292	Forward primer: RH11	a	Total volume 25 μl	[18]
			5’-CATCCGGTCGATCCTGCC-3’			
			Reverse primer: RH4	96°C, 45 s	d	
			5’-AGTCGAACCCTGATTCTCCGCCAGG-3’	50°C, 30 s	0.15 μl Taq-Ti hot start DNA polymerase^e^	
				72°C, 45 s		
				→ 35 cycles	5% dimethyl sulfoxide (DMSO)^f^	
				b		
	Secondary reaction	130	Forward primer: GiarF	a	2 μl from the 1st-round PCR reaction	[19]
			5’-GACGCTCTCCCCAAGGAC-3’			
			Reverse primer: GiarR	96°C, 45 s		
			5’-CTGCGTCACGCTGCTCG-3’	55°C, 30 s		
				72°C, 45 s		
				→ 35 cycles		
				b		
β-giardin	Primary reaction	753	Forward primer: G7	a	Total volume 25 μl	[20]
			5’-AAGCCCGACGACCTCACCCGCAGTGC-3’			
			Reverse primer: G759	95°C, 30 s	d	
			5’-GAGGCCGCCCTGGATCTTCGAGACGAC-3’	50°C, 30 s	0.15 μl Tth Plus DNA polymerase^e^	
				72°C, 60 s		
				→ 40 cycles		
				b		
	Secondary reaction	511	Forward primer: B-F	a	2 μl from the 1st-round PCR reaction	[21]
			5’-GAACGAACGAGATCGAGGTCCG-3’			
			Reverse primer: B-R	96°C, 45 s		
			5’-CTCGACGAGCTTCGTGTT-3’	55°C, 30 s		
				72°C, 45 s		
				→ 35cycles		
				b		
GDH	Primary reaction	not given	Forward primer: GDHeF	c	Total volume 25μl	[19]
			5’-TCAACGTYAAYCGYGGYTTCCGT-3’			
			Reverse primer: GDHiR	94°C, 30 s	d	
			5’-GTTRTCCTTGCACATCTCC-3’	50°C, 30 s	0.2 μl Tth Plus DNA polymerase^e^	
				72°C, 60 s		
				→ 40 cycles		
				b		
	Secondary reaction	432	Forward primer: GDHiF	c	2 μl from the 1st-round PCR reaction	[19]
			5’-CAGTACAACTCYGCTCTCGG-3’			
			Reverse primer: GDHiR	94°C, 30 s		
			5’-GTTRTCCTTGCACATCTCC-3’	60°C, 30 s		
				72°C, 60 s		
				→ 40 cycles		
				b		

a: Initial activation step: 96°C, 5 min.

b: Final extension: 72°C, 7 min.

c: Initial activation step: 94°C, 5 min.

d: used substances: 2 μl diluted DNA template, 2.5 μl 10x Reaction Buffer , 2.5 μl MgCl_2_ (25 mM), 1 μl dNTPs (5 mM) (Promega), 1 μl of each primer (10 μM), Water-ultra pure grade (Fisher Biotech Perth, Australia).

e: Fisher Biotech Perth, Australia.

f: Sigma–Aldrich St. Louis, Missouri.

#### DNA sequencing

PCR products were purified using Agencourt AMPure XP magnetic beads (Beckman coulter, Beverly, USA) as per the manufacturer’s instructions. Sequence reactions were performed using the Big Dye Terminator Version 3.1 cycle sequencing kit (Applied Biosystems) according to the manufacturer’s instructions. PCR products were sequenced with the second round primers (1 μl [2.5 μM]). The cycling conditions for nucleotide sequencing are: 1 cycle of 96°C for 2 min and 25 cycles at 96°C for 10 s, 50°C for 5 s and 60°C for 4 min. Reactions were electrophoresed on an ABI 3730 48 capillary machine.

#### Species identification

Sequences were analysed using Sequencher 4.8 (Gene Codes, Ann Arbor, MI, USA) and compared to published sequences (Table 2) to identify species and sub-genotype information.

**Table 2:** **GenBank accession numbers used for alignment with *Giardia *** **sequences**

** 18S rRNA**		** β-giardin**		** GDH**	
AI	AF199445	A1	X14185	A	DQ100288
AI	M54878	A2	AY545645	A	M84604
AII	AF199446	A2	FN386482	A1	DQ414242
AIII	AF199447	A5	AY545643	A2	L40510
B	U09491	A8	AY545649	B	AY826193
B	U09492	B	AY072728	B3	AF069059
C	AF199449	B	AY647266	B4	AY178750
D	AF199443	C	AY545646	C	U60982
E	AF199448	C	FJ009206	D	U60986
E	DQ157272	D	AY545648	E	AY178741
F	AF199444	E	EU189375	F	AF069057
G	AF199450	E1	AY072729	G	AF069060
		E2	AY545650		
		E3	AY653159		

## Results

Of the 152 samples, diagnosed *Giardia-*positive by microscopy, 110 (72.4%) were positive by PCR and successfully sequenced.

Sequence analysis identified the presence of *G. bovis* (Assemblage E) in 101/110 (91.8%) PCR-positive samples, *G. duodenalis* (Assemblage A) in 8/110 (7.3%) samples and a mixed template of *G. duodenalis* and *G. bovis* (Assemblage A+E) in 1/110 (0.9%) samples. Using the β-giardin and GDH genes it was possible to identify sub-genotypes within the species *G. bovis* (E2 and E3) and *G. duodenalis* (A1) (Table 3).

**Table 3:** **Genotypic characterisation of *Giardia *** **spp. isolates at different loci**

**18S rRNA**	**β-giardin**	**GDH**	**18S rRNA and β-giardin**	**18S and GDH**	**18S rRNA, β-giardin and GDH**
A (5)	A1 (1)	A1 (1)	E, E (1)	E, A1 (1)	A, A1, A (1)
E (85)	E3 (1)	E (1)	E, E2 (1)	E, E (1)	E, E3, E (3)
			E, E3 (8)		

Of the 110 PCR-positive samples 94 (85.5%) samples amplified at one locus, whereas 12/110 (10.9%) and 4/110 (3.6%) samples amplified at 2 and 3 loci, respectively. 18S amplified most frequently (106/152 samples, 69.7%), whereas β-giardin and GDH amplified comparatively rarely (16/152, 10.5%; 8/152, 5.3%) (Table 3).

Table 4 shows that in the majority of the calves with diarrhoea a co-infection with *Cryptosporidium* spp. or *Eimeria* spp. was present.

**Table 4:** **Distribution of mono- and mixed infections of *Giardia*-positive calves in relation to faecal consistency**

		**Total**	**Monoinfection with *Giardia *** **spp.**	**Coinfection with *Cryptosporidium *** **spp.**	**Coinfection with *Eimeria *** **spp.**
MIFC positive	Total	152	66	15	71
	With diarrhoea	62	25	10	27
	Without diarrhoea	90	41	5	44
PCR: *G. duodenalis*	Total	8	-	3	5
	With diarrhoea	4	-	2	2
	Without diarrhoea	4	-	1	3
PCR: *G. bovis*	Total	101	48	8	45
	With diarrhoea	38	17	6	15
	Without diarrhoea	63	31	2	30
PCR: *G. duodenalis + G. bovis*	Total	1	1	-	-
	With diarrhoea	-	-	-	-
	Without diarrhoea	1	1	-	-

### Discussion

The results of this study reveal that the livestock-specific species *G. bovis* (Assemblage E) is the most frequent species (91.8%) in calves in Southern Germany. The zoonotic species *G. duodenalis* (Assemblage A) was found in a low number of samples (7.3%), while a mixed infection of *G. duodenalis* and *G. bovis* was identified in only one sample (0.9%). *G. enterica* (Assemblage B), the second zoonotic species, was not detected in this study.

Similarly in another study on German calves, the same species were detected and *G. bovis* was also found most frequently; however, there was a higher proportion of infection with *G. duodenalis* as well as with mixed infections than observed in this study [15].

Finding *G. bovis* in the majority of *Giardia*-infections in calves and *G. duodenalis* in only some cases also concurs with the results of former studies on cattle [10-12,22-24]. In some studies *G. bovis* was the only species identified in calves [9,25]. *G. enterica* was not detected in this study, which is in accordance with the results of many previous studies although several did find this genotype in cattle [10,12,13,21]. One study diagnosed *G. enterica* more frequently than *G. bovis*[26] whereas studies in New Zealand found only infections with *G. duodenalis* and *G. enterica,* but not with *G. bovis*[27-29].

The finding of sub-genotypes E2 and E3 within the species *G. bovis* (Assemblage E) is similar to former studies [11,14,21]. According to Xiao and Fayer [30] and Feng and Xiao [1] A1 and A2 are the most common sub-genotypes of *G. duodenalis* (Assemblage A), with humans being mostly infected with A2 and animals with A1. This agrees with former results [14,22,23] and with the results of this study, as A1 was the only sub-genotype of *G. duodenalis* diagnosed. However, others have found one or more of the sub-genotypes A1-A4 in cattle [10-12,21,24]. Therefore it is possible that calves can be infected with a variety of sub-genotypes of *G. duodenalis*, all of which have also been identified in humans [21]. This suggests that there may be an interaction between the human and livestock transmission cycle [3]. Cattle have long been assumed to be of public health significance as a source of waterborne outbreaks of giardiasis in humans due to contamination of ground and surface water, although, there is no evidence incriminating infected cattle in any of the 132 documented waterborne outbreaks [2]. However, it has been shown, that animal handlers can be in danger of zoonotic transmission of *G. duodenalis* from infected cattle [14], and in reverse anthropozoonotic transmission of *G. duodenalis* from animal handlers to cattle is also possible [13]. Thus, transmission of the zoonotic species, which was detected in this study, could in principle be possible between animal handlers and cattle.

The role of *Giardia* as a cause of diarrhoea in calves is still unclear, as there are conflicting results from a number of studies, some demonstrating an association and others not. Furthermore, the presence of species-specific pathogenicity in calves poses further difficulties in the evaluation and has not been determined in another bovine study [11]. The role of the particular *Giardia*-species in mixed-infections in diarrhoeic calves could not be clarified either. However, the identification of some diarrhoeic samples, where *G. bovis* was the only pathogen detected, may suggest that this species does contribute to diarrhoea in calves. Whether these results are indicative or not remains unclear. Further studies will show whether differences in the clinical outcomes can occur due to the various sub-genotypes as has been established in human medicine [2].

### Conclusions

The results of this study show that although the livestock specific species *G. bovis* has been diagnosed most frequently, the potential zoonotic species *G. duodenalis* is also present in calves in Southern Germany and thus might be a risk for animal handlers. Furthermore the results indicate that *G. bovis* might contribute to diarrhoea, as it was the only pathogen found in a proportion of the samples from diarrhoeic calves.

## Competing interests

The authors declare that they have no competing interests.

## Authors’ contributions

JG prepared the samples, analysed and interpreted the data and drafted the manuscript, AA and LP carried out the PCR and the sequence analysis, AT participated in the design and conception of the study and reviewed the draft, KP and MS conceived of the study, participated in its design and conception and helped to draft the manuscript. All authors read and approved the final manuscript.
